# Global Patterns of Protein Domain Gain and Loss in Superkingdoms

**DOI:** 10.1371/journal.pcbi.1003452

**Published:** 2014-01-30

**Authors:** Arshan Nasir, Kyung Mo Kim, Gustavo Caetano-Anollés

**Affiliations:** 1 Evolutionary Bioinformatics Laboratory, Department of Crop Sciences, University of Illinois, Urbana, Illinois, United States of America; 2 Illinois Informatics Institute, University of Illinois, Urbana, Illinois, United States of America; 3 Microbial Resource Center, Korea Research Institute of Bioscience and Biotechnology, Daejeon, Korea; University College London, United Kingdom

## Abstract

Domains are modules within proteins that can fold and function independently and are evolutionarily conserved. Here we compared the usage and distribution of protein domain families in the free-living proteomes of Archaea, Bacteria and Eukarya and reconstructed species phylogenies while tracing the history of domain emergence and loss in proteomes. We show that both gains and losses of domains occurred frequently during proteome evolution. The rate of domain discovery increased approximately linearly in evolutionary time. Remarkably, gains generally outnumbered losses and the gain-to-loss ratios were much higher in akaryotes compared to eukaryotes. Functional annotations of domain families revealed that both Archaea and Bacteria gained and lost metabolic capabilities during the course of evolution while Eukarya acquired a number of diverse molecular functions including those involved in extracellular processes, immunological mechanisms, and cell regulation. Results also highlighted significant contemporary sharing of informational enzymes between Archaea and Eukarya and metabolic enzymes between Bacteria and Eukarya. Finally, the analysis provided useful insights into the evolution of species. The archaeal superkingdom appeared first in evolution by gradual loss of ancestral domains, bacterial lineages were the first to gain superkingdom-specific domains, and eukaryotes (likely) originated when an expanding proto-eukaryotic stem lineage gained organelles through endosymbiosis of already diversified bacterial lineages. The evolutionary dynamics of domain families in proteomes and the increasing number of domain gains is predicted to redefine the persistence strategies of organisms in superkingdoms, influence the make up of molecular functions, and enhance organismal complexity by the generation of new domain architectures. This dynamics highlights ongoing secondary evolutionary adaptations in akaryotic microbes, especially Archaea.

## Author Summary

Proteins are made up of well-packed structural units referred to as domains. Domain structure in proteins is responsible for protein function and is evolutionarily conserved. Here we report global patterns of protein domain gain and loss in the three superkingdoms of life. We reconstructed phylogenetic trees using domain fold families as phylogenetic characters and retraced the history of character changes along the many branches of the tree of life. Results revealed that both domain gains and losses were frequent events in the evolution of cells. However, domain gains generally overshadowed the number of losses. This trend was consistent in the three superkingdoms. However, the rate of domain discovery was highest in akaryotic microbes. Domain gains occurred throughout the evolutionary timeline albeit at a non-uniform rate. Our study sheds light into the evolutionary history of living organisms and highlights important ongoing mechanisms that are responsible for secondary evolutionary adaptations in the three superkingdoms of life.

## Introduction

Proteins are biologically active molecules that perform a wide variety of functions in cells. They are involved in catalytic activities (e.g. enzymes), cell-to-cell signaling (hormones), immune response initiation against invading pathogens (antibodies), decoding genetic information (transcription and translation machinery), and many other vital cellular processes (receptors, transporters, transcription factors). Proteins carry out these functions with the help of well-packed structural units referred to as domains. Domains are modules within proteins that can fold and function independently and are evolutionarily conserved [1]–[4]. It is the domain make up of the cell that defines its molecular activities and leads to interesting evolutionary dynamics [5].

Different mechanisms have been described to explain the evolution of domain repertoires in cells [3]. These include the reuse of existing domains [2], [6], interplay between gains and losses [7]–[9], *de novo* domain generation [1], and horizontal gene transfer (HGT) [10]. Domains that appeared early in evolution are generally more abundant than recently emerged domains and can be reused in different combinations in proteins. This recruitment of ancient domains is an ongoing evolutionary process that leads to the generation of novel domain architectures (i.e. ordering of domains in proteins) by gene fusion, exon recombination and retrotransposition [2]–[4], [11]. For example, aminoacyl-tRNA synthetases are enzymes that charge tRNAs with ‘correct’ amino acids during translation [12], [13]. These crucial enzymes are multidomain proteins that encode a catalytic domain, an anticodon-binding domain, and in some cases, accessory domains involved in RNA binding and editing [13]. Evolutionary analysis suggests that these domains were recruited gradually over time [14]. In fact, recruitment of ancient domains to perform new functions is a recurrent phenomenon in metabolism [15].

In addition to the frequent reuse of domains, the dynamics between gains and losses also impacts the evolution of proteome repertoires [7]–[9]. Previous studies identified high rates of gene gains and losses in 12 closely related strains of *Drosophila* [7], *Prochlorococcus* (a genus of cyanobacteria) [16], and 60 isolates of *Burkholderia* (a genus of proteobacteria) [17]. A recent analysis of Pfam domains [18] revealed that ∼3% of the domain sequences were unique to primates and had emerged quite recently [19]. This implies that emergence of novel domains is an incessant evolutionary process [1]. In contrast, different selective pressures can lead to loss of domains in certain lineages and trigger major evolutionary transitions. For example, the increased rate of domain loss has been linked to reductive evolution of the proteomes of the archaeal superkingdom [20], adaptation to parasitism in cells [21] (e.g. transition from the free-living lifestyle to obligate parasitism in *Rickettsia* [22]), and ‘de-evolution’ of animals [23], [24] from their common ancestor. In these studies, gain and loss inferences were restricted to only particular groups of phyla or organisms. A global analysis involving proteomes from the three superkingdoms remained a challenge. Finally, changes to domain repertoires are also possible by HGT that is believed to occur with high frequency in microbial species, especially Bacteria [25], [26].

Here, we describe the evolutionary dynamics of protein domains grouped into fold families (FFs) and model the effects of domain gain and loss in the proteomes of 420 free-living organisms that have been fully sequenced and were carefully sampled from Archaea, Bacteria, and Eukarya (Dataset S1). The 420-proteome dataset was previously used by our group to reconstruct the evolutionary history of free-living organisms (see [27]) and was updated here to account for recent changes in protein classification and functional annotation. The dataset is very well annotated, especially regarding organism lifestyles that are otherwise problematic to assign, has already produced patterns of protein and proteome evolution that are very useful (including those described in [27]), and has produced timelines of FF evolution that are being actively mined. We conducted phylogenomic analyses using the *abundance* (total redundant number of each FF in every proteome) [28], [29] and *occurrence* (presence or absence) [30], [31] counts of FFs as phylogenetic characters to distinguish the 420 sampled taxa (i.e. proteomes). FF information was retrieved from the Structural Classification of Proteins (SCOP) database, which is considered a ‘gold standard’ for the classification of protein domains into different hierarchical levels [32]. Current SCOP definitions group protein domains with high pair-wise sequence identity (>30%) into a common FF, FFs that are evolutionarily related into fold superfamilies (FSFs), FSFs with similar secondary structure arrangement into folds (Fs), and Fs with common secondary structure elements into a handful of protein classes [33], [34]. A total of 110,800 SCOP domains (ver. 1.75) are classified into a finite set of only 1,195 Fs, 1,962 FSFs and 3,902 FFs. The lower number of distinct FSFs and FFs suggests that domain structure is far more conserved than molecular sequence (e.g. see [35]) and is reliable for phylogenetic studies involving the systematic comparison of proteomes [27]. Another advantage of using SCOP domains is the consideration of known structural and inferred evolutionary relationships in classifying domains into FFs and FSFs [36]. In comparison, evolutionary relationships for the majority of the Pfam domains are unknown. We further restricted the analysis to include only FF domains as they are conserved enough to explore both the very deep and derived branches of the tree of life (ToL) and are functionally orthologous [37]. In contrast, FSF domains represent a higher level in SCOP hierarchy and are more conserved than FFs but may or may not be functionally orthologous. Moreover, high conservation of FSF domains is useful for exploring the deep branches of the ToL but may not be very informative for the more derived relationships.

The analysis of retracing the history of changes in the occurrence and abundance of FF domains on each branch of the reconstructed ToLs revealed that FFs were subject to high rates of gains and losses. Domain gains generally outnumbered losses but both occurred with high frequencies throughout the evolutionary timeline and in all superkingdoms. Remarkably, the gains-to-loss ratios increased with evolutionary time and were relatively higher in the late evolutionary periods. Finally, functional annotations of FFs illustrated significant differences between superkingdoms and described modern tendencies in proteomes.

## Methods

### Data retrieval and processing

The 420-proteome dataset used in this study included proteomes from 48 Archaea, 239 Bacteria, and 133 Eukarya. The dataset did not include any parasitic organisms as they harbor reduced proteomes and bias the global phylogenomic analyses (e.g. [38]). FFs were assigned to proteomes using SUPERFAMILY ver. 1.73 [39] hidden Markov models [40], [41] at an *E*-value cutoff of 10^−4^ [42]. A total of 2,397 significant FF domains were detected in the sampled proteomes. The definitions of eight FFs in the 420-proteome dataset were updated in SCOP ver. 1.75 and were therefore renamed in our dataset. FFs were referenced using SCOP *concise classification strings (css)* (e.g. ‘Ferredoxin reductase FAD-binding domain-like’ FF is b.43.4.2, where b represents the class [all-beta proteins], 43 the fold, 4 the FSF and 2 the FF).

### Phylogenomic analysis

We considered the genomic *abundance* [28], [29] and *occurrence* [30], [31] of 2,397 FFs as phylogenetic characters to reconstruct phylogenies describing the evolution of 420 free-living organisms (i.e. taxa) using maximum parsimony. The raw abundance values of each FF in every proteome (*g_ab_*) were log-transformed and divided by the logarithm of maximum value in the matrix (*g_max_*) to account for unequal proteome sizes and variances (see formula below) [29], [43].
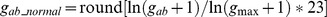
The transformed abundance values were then rescaled from 0 to 23 (scaling constant) in an alphanumeric format (0–9 and A-N) to allow compatibility with the phylogenetic reconstruction software. The transformed abundance matrix with 24 possible character states was imported into PAUP* 4.0b10 [44] for the reconstruction of *abundance* trees. For *occurrence* trees, we simply used 0 and 1 (indicating absence and presence) as the valid character state symbols. We polarized both *abundance* and *occurrence* trees using the ANCSTATES command in PAUP* and designated character state 0 as the ancestral state, since the most ancient proteome is closer to a simple progenote organism that harbors only a handful of domains [20], [38]. The stem lineage of this organism gradually increased its domain repertoire, supporting the polarization from 0 to N and Weston's generality criterion, in which the taxic distribution of a set of character states is a subset of the distribution of another [45], [46]. Phylogenetic trees are adequately interpreted when rooted. This provides direction to the flow of evolutionary information and is useful to study species adaptations. In this study, we choose to root trees using the Lundberg method [47]. This scheme first determines the most parsimonious unrooted tree, which is then attached to a hypothetical ancestor. The hypothetical ancestor may be attached to any of the branches in the tree. However, only the branch that gives the minimum increase in overall tree length is selected [48]. This branch, which exhibits the largest numbers of ancestral (plesiomorphic) character states was specified using the ANCSTATES command in PAUP*. Thus, Lundberg rooting automatically roots the trees by preserving the principle of maximum parsimony. This method is simple and free from artificial biases introduced by alternative rooting methods (e.g. the outgroup method). While selection of an appropriate outgroup to root the ToL is virtually impossible, Lundberg rooting provides a parsimonious estimate of the overall phylogeny and should be considered robust as long as the assumptions used to root the trees are not proven false. To evaluate support for the deep branches of ToLs, we ran bootstrap (BS) analysis with 1,000 replicates. Character state changes were recorded by specifying the ‘chglist’ option in PAUP*. Trees were visualized using Dendroscope ver. 3.0.14b [49].

### Tree comparison

To determine congruence between *abundance* and *occurrence* trees, we used the nodal module implemented in the TOPD/FMTS package ver. 3.3 [50]. The module takes as input a set of trees in Newick format and calculates a root mean squared deviation (RMSD) value for each pairwise comparison. The RMSD value is 0 for identical trees and increases with incongruence. To evaluate the significance of calculated RMSD values, we implemented the ‘Guided randomization test’ with 100 replications to determine whether the calculated RMSD value was smaller than the chance expectation. The randomization test randomly changes the positions of taxa in trees, while maintaining original tree topology, and calculates an RMSD value for each random comparison [50]. The result is a random distribution of RMSD values with a mean and standard deviation. The calculated RMSD value was compared with the mean of the random distribution to determine whether the observed differences were better than what would be expected merely by chance.

### Spread (popularity) of FFs in proteomes

The spread of each FF was given by its distribution index (*f*-value), defined by the total number of proteomes encoding a particular FF divided by the total number of proteomes. The *f*-value ranges from 0 (absence from all proteomes) to 1 (complete presence).

### Molecular and geological age of FFs

To determine the relative age of FF domains in our dataset, we reconstructed trees of domains (ToDs) from the *abundance* and *occurrence* matrices used in the reconstruction of ToLs. The matrices were transposed, treating FFs as taxa and proteomes as characters. The reconstructed ToDs described the evolution of domains grouped into FFs and identified the most ancient and derived FFs (refer to [27] for an elaborate description and discussion on ToDs). To root the trees, we declared character state ‘N’ as the most ancestral state. This axiom of polarization considers that history of change for the most part obeys the ‘principle of spatiotemporal continuity’ (sensu Leibnitz) that supports the existence of Darwinian evolution. Specifically, it considers that abundance and diversity of individual FFs increases progressively in nature by gene duplication (and associated processes of subfunctionalization and neofunctionalization) and *de novo* gene creation, even in the presence of loss, lateral transfer or evolutionary constraints in individual lineages. Consequently, ancient domains have more time to accumulate and increase their abundance in proteomes. In comparison, domains originating recently are less popular and are specific to fewer lineages. We note that the N to 0 polarization is supported by the observation that FFs that appear at the base of the ToDs are structures that are widespread in metabolism and are considered to be of very ancient origin (e.g. [27]). The age of each FF was drawn directly from the ToDs using a PERL script that calculates the distance of each node from the root. This node distance (*nd*) is given on a relative scale and portrays the origin of FFs from 0 (most ancient) to 1 (most recent). The geological ages of FFs were derived from a molecular clock of protein folds [51], [52] that was used to calibrate important events in proteome evolution. We have previously shown that *nd* correlates with geological time, following a molecular clock that can be used as a reliable approximation to date the appearance of protein domains [51], [52].

### Functional annotations

We used the SUPERFAMILY functional annotation scheme (based on SCOP 1.73) to study the functional roles of FF domains in our dataset [53]–[55]. The SUPERFAMILY annotation assigns a single molecular function to FSF domains (and by extension to its descendant FFs). The annotation scheme gives a simplified view of the functional repertoire of proteomes using seven major functional categories including, i) *metabolism*, ii) *information*, iii) *intracellular processes*, iv) *extracellular processes*, v) *general*, vi) *regulation* and vii) *other* (includes domains with either unknown or viral functions). We assumed that FFs grouped into an FSF performed the same function that was assigned to their parent FSF. While this simplistic representation does not demonstrate the complete functional capabilities of a cell, it is sufficient to illustrate the major functional preferences in proteomes (refer to [21] for further description and use of the functional annotation scheme in large-scale proteomic studies).

### Gene Ontology (GO) enrichment analysis

We conducted a GO enrichment analysis [56], [57] on FF domains to identify biological processes [58], [59] that were significantly enriched. For this purpose, the list of FF domains was given as input to domain-centric Gene Ontology (dcGO; http://supfam.org/SUPERFAMILY/dcGO) resource and the most specific and significant associations to GO terms corresponding to different biological processes were retrieved. The statistical significance was evaluated by *P*-value computed under the hypergeometric distribution [56], while the false discovery rate (FDR) was set to default at <0.01 [60].

## Results

We first describe the patterns of FF use and reuse in superkingdoms and then build on this knowledge to infer the meanings of domain gain and loss in proteomes.

### Evolutionary history of FF domains

**Figure 1:**
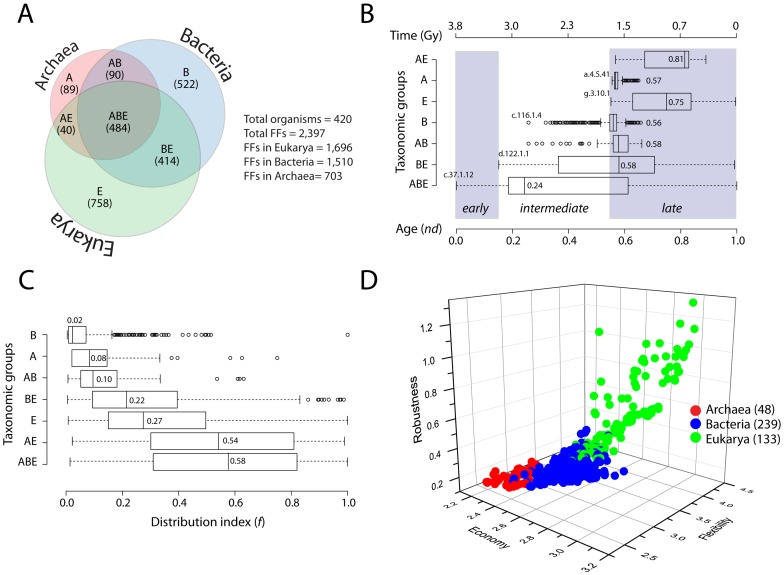
Evolutionary dynamics of FFs and organismal persistence strategies. A) A Venn diagram describes the distribution of FFs in the seven taxonomic groups (reproduced from [27]) B) Boxplots represent the distributions of domain ages (*nd*) for each taxonomic group. Numbers within each distribution indicate group medians, hollow circles the outliers, while the shaded regions identify important evolutionary epochs. Geological time (Gy) was inferred from a molecular clock of protein folds [51], [52]. FFs were identified by SCOP *css*: c.37.1.12, ABC transporter ATPase domain-like; d.122.1.1, Heat shock protein 90, HSP 90, N-terminal domain; c.116.1.4, tRNA(m1G37)-methyltransferase TrmD; g.3.10.1, Colipase-like; a.4.5.41, Transcription factor E/IIe-alpha, N-terminal domain. C) Boxplots represent the distribution index (*f*-value) of FF domains for each taxonomic group. Numbers within each distribution indicate group medians. Hollow circles represent outliers. D) A 3D scatter plot describes the persistence strategies of Archaea (red), Bacteria (blue), and Eukarya (green). All axes are in logarithmic scale. Numbers in parenthesis indicate total number of proteomes available for study in each superkingdom.

A Venn diagram describes the sharing patterns of 2,397 FFs in seven Venn distribution groups (Figure 1A). For simplicity, we name these sets ‘taxonomic groups’ with the understanding that their taxonomic status is endowed by patterns of distribution of FFs in superkingdoms. The number of FFs decreased in the order Eukarya (total FFs = 1,696), Bacteria (1,510) and Archaea (703). Eukarya also had the highest number of superkingdom specific FFs (758), followed by Bacteria (522), and Archaea (89). ABE FFs were universal (i.e. present in all three superkingdoms) and made the third largest group with 484 FFs, while BE was the fourth largest taxonomic group with 414 FFs (Figure 1A). The lowest number of FFs was in AE with only 40 FFs that were unique to both Archaea and Eukarya. The number of Archaea-specific FFs was also low (89) but comparable to the number of akaryotic FFs (i.e. AB = 90). We observed that Archaea was mostly about sharing (or not innovating new FFs). This was evident by the fact that only 13% of the total archaeal FFs were Archaea-specific. This was in striking contrast with Bacteria and Eukarya where superkingdom-specific FFs made large proportions of the FF repertoires with 35% and 45% FFs, respectively (Figure 1A).

We plotted the distribution of domain ages (*nd*) for FFs in each taxonomic group to determine the order of their evolutionary appearance (Figure 1B) (see Methods). The first FF to appear in evolution was the ‘ABC transporter ATPase domain-like’ (c.37.1.12) FF at *nd* = 0 in the ABE taxonomic group (Figure 1B). ABC transporters are multifunctional proteins that are primarily involved in the transport of various substrates across membranes [61], [62]. These domains are ubiquitous and highly abundant in extant species and considered to be very ancient. In our timeline, c.37.1.12 appeared first, supporting its widespread presence and significance in cells. ABE was the most ancient taxonomic group spanning the entire time axis with a median *nd* of 0.24 (Figure 1B). This suggested that the majority of the FFs that were common across all superkingdoms appeared very early in evolution. ABE was followed by the appearances of BE (at *nd* = 0.15), AB (0.26), B (0.26), E (0.551), A (0.555), and AE (0.57) taxonomic groups, in that order (Figure 1B).

The first complete loss event for any FF in the primordial world likely triggered the appearance of the BE taxonomic group. Our data indicates that this occurred at *nd* = 0.15 (roughly >3.2 billion [Gyrs] years ago) with the complete loss of the ‘Heat shock protein 90, HSP90, N-terminal domain’ (d.122.1.1) FF in Archaea (Figure 1B). Heat-shock proteins are molecular chaperones that assist in protein folding and clearing of cell debris [63]. These are highly conserved in bacterial and eukaryal species, but relatively less abundant in Archaea. In fact, homologs of Hsp90 or Hsp100 are completely absent in archaeal species [63]. This knowledge is compatible with our finding of loss of d.122.1.1 FF in Archaea that occurred very early in evolution. We propose that this event exemplifies reductive evolutionary processes that were at play early in evolution in nascent archaeal lineages as emergent diversified cells were unfolding different mechanisms of protein folding. In light of our results, Archaea was the first superkingdom to follow reductive trends. The first superkingdom-specific FF appeared in B at *nd* = 0.26 (∼2.8 Gyrs ago), while both Archaea and Eukarya acquired unique FF domains concurrently at around *nd* = 0.55 (∼1.6 Gyrs ago) (Figure 1B). Emergence of taxonomic groups in evolution described three important evolutionary epochs: (i) *early* (0≤*nd*<0.15), a period before the start of reductive evolution in the archaeal superkingdom, (ii) *intermediate* (0.15≤*nd*<0.55), a period marked by early domain discovery in Bacteria, and (iii) *late* (0.55≤*nd*≤1), a period during which simultaneous diversification of Archaea and Eukarya occurred (Figure 1B).

To determine the popularity of FFs across organisms, we computed an *f*-value representing the fraction of proteomes encoding an FF. The median *f*-value decreased in the order, ABE>AE>E>BE>AB>A>B (Figure 1C). We observed that universal FFs of the ABE taxonomic group were most popular and shared by the majority of the proteomes (median *f* = 0.58). The FFs in AE and E were also distributed with higher *f*-values (median *f* = 0.54 and 0.27). In contrast, most of the bacterial taxonomic groups (e.g. BE, AB and B) had lower median *f*-values (0.22, 0.10, and 0.02, respectively). The Venn diagram indicated that ∼22% of the total FFs were bacteria-specific (Figure 1A) but the median *f*-value of those FFs was extremely low (0.02) (Figure 1C). This implied that FFs unique to Bacteria were very unevenly distributed among bacterial species. This also suggested that the rate of FF discovery in Bacteria was very high but their spread was quite limited.

A recent study proposed concepts of economy (i.e. organism budget in terms of number of unique genes and domain structures), flexibility (potential of an organism to adapt to environmental change) and robustness (ability to resist damage and change) to help explain the persistence strategies utilized by organisms in the three superkingdoms [64]. To determine how persistence strategies distributed in our dataset, we redefined economy (i.e. total number of unique FFs in a proteome), flexibility (total number of redundant FFs in a proteome) and robustness (ratio of flexibility to economy). When plotted together on a 3D plot, interesting patterns were revealed (Figure 1D). As expected, the proteomes of the akaryotic microbes in Archaea and Bacteria were most economical but least flexible and robust (Figure 1D). Within these superkingdoms, archaeal proteomes (red circles) exhibited greatest economy but lowest flexibility and robustness. In contrast, Bacteria exhibited intermediate levels of economy, flexibility and robustness. Finally, eukaryal proteomes were least economical but highly flexible and robust (Figure 1D). Table 1 lists the lower and upper bounds for economy, flexibility, and robustness for the three superkingdoms. The median values for the three parameters always increased in the order, Archaea, Bacteria, and Eukarya (Table 1). The analysis revealed that the survival strategy of microbial species lies in encoding smaller domain repertoires while the eukaryal species trade-off economy with more flexibility and robustness and harbor richer proteomes [64]. The number of both unique (economy) and redundant FFs (flexibility and robustness) was considerably higher in eukaryotes.

**Table 1:** Descriptive statistics on the total number of proteomes (*N*), minimum (*min*), maximum (*max*) and median values for raw counts of occurrence, abundance and ratio of FFs in each superkingdom.

Occurrence					Abundance			Ratio		
Superkingdom	*N*	*Min*	*max*	*median*	*min*	*Max*	*median*	*Min*	*max*	*median*
Archaea	48	174^1^	293^2^	236	264^1^	598^2^	377.5	1.46^3^	2.10^4^	1.64
Bacteria	239	239^5^	824^6^	426	376^5^	1958^7^	883	1.52^8^	3.40^9^	1.98
Eukarya	133	364^10^	1089^11^	674	982^12^	19917^13^	2875	2.24^12^	20.41^13^	4.04

The superscripts identify individual species.

1 *Staphylothermus marinus,*

2 *Methanosarcina acetovirans,*

3 *Thermoplasma volcanium,*

4 *Haloarcula marismortui,*

5 *Dehalococcoides sp.,*

6 *Citrobacter koseri,*

7 *Burkholderia xenovorans,*

8 *Nitratiruptor sp.,*

9 *Rhodococcus sp.,*

10 *Paramecium tetraurelia,*

11 *Homo sapiens,*

12 *Malassezia globosa,*

13 *Takifugu rubripes.*

### Functional annotation of FF domains in history

**Figure 2:**
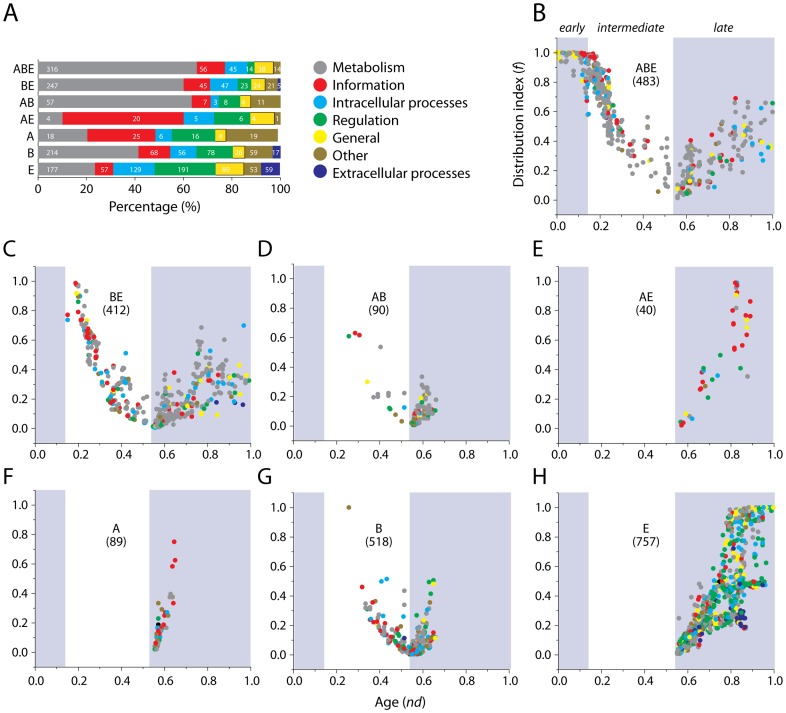
Functional annotation of FF domains. A) Stacked bar plots describe the distribution of molecular functions in each of the seven taxonomic groups. The size of each bar is proportional to the percentage of FF domains in each functional category, while the numbers indicate total counts of FFs annotated in that category. B–H) Scatter plots illustrate the emergence of molecular functions in taxonomic groups. The *x*-axes represents evolutionary time (*nd*), while the *y*-axes indicate the distribution index (*f*-value) of FFs. Evolutionary epochs identified as previously. Numbers in parenthesis indicate total number of FF domains in each taxonomic group for which SUPERFAMILY functional annotations (based on SCOP 1.73) were available.

We compared the distributions of molecular functions in taxonomic groups (Figure 2A) and dated their appearance in evolutionary time (*nd*) (Figure 2B–H). *Metabolism* was the most abundant and widely distributed molecular function in organisms, especially in the ABE, BE, and AB taxonomic groups. However, significant deviations were observed in the AE and A taxonomic groups, where informational FFs (e.g. those belonging to the replication machinery) outnumbered FFs in other functional categories (Figure 2A). These results are consistent with previous knowledge regarding high sharing of informational proteins between Archaea and Eukarya and a common metabolic apparatus between Bacteria and Eukarya. This observation has often led to proposals relating the origin of eukaryotes to a confluence between akaryotic cells (reviewed in [65]; see also [66]–[69]). However, our data show that the presence of bacterial metabolic enzymes in Eukarya is better explained by primordial endosymbiotic events leading to mitochondria and plastids in a proto-eukaryote stem cell-line (read below). In comparison, sharing of informational enzymes between Archaea and Eukarya occurred relatively late in evolution and could actually reflect late domain losses in Bacteria. *Intracellular processes* and *general* were distributed similarly while *regulation* and *extracellular processes* appeared to be preferential only in Eukarya (Figure 2A). The distribution of molecular functions in taxonomic groups was largely in agreement with the distribution previously explained for individual species [21].

We explored the order of evolutionary appearance of molecular functions by generating *nd* vs. *f* plots for the seven taxonomic groups (Figure 2B–H). The ABE FFs were present with largest *f*-values and as expected spanned the entire *nd*-axis (Figure 2B). In fact, 13 FFs had an *f*-value of 1.0 indicating universal presence in organisms, while 62 near-universal FFs were present in >95% of the proteomes. ABE FFs were generally enriched in metabolic functions (Figure 2B). This suggested that the last common ancestor of diversified life was structurally and metabolically versatile (e.g. [38]). However, the *f*-value distribution of ABE FFs followed a bimodal pattern with a significant drop in *f* during the *intermediate* evolutionary epoch. Most of the FFs of intermediate age were classified as metabolic (grey circles), informational (red circles), or with intracellular roles (light blue circles) (Figure 2A, 2B). BE followed a distribution similar to ABE but the first FF appeared during the *intermediate* evolutionary epoch at *nd* = 0.15 (Figure 2C). This also marked the first loss of an FF in Archaea (boxplot for BE in Figure 1B). This observation implies that Archaea was the first superkingdom to escape from the ancestral community and evolved by streamlining genomes. Perhaps, genome reduction was better suited for harsher environments. Other selective pressures that may have triggered early domain loss in Archaea could include escape from RNA viruses (because RNA is unstable at extreme temperatures) and phagotrophs [70]. The majority of the BE FFs served metabolic, informational and intracellular roles (Figure 2A, 2C), just like ABE. The akaryotic-specific (AB) FFs appeared during the *intermediate* and *late* evolutionary epochs and were largely dominated by metabolic and *other* FFs (Figure 2A, 2D). Most of these FFs had very low *f*-values (Figure 2D) indicating that this taxonomic group exhibited low popularity levels. In contrast, all of the 40 AE FFs appeared in the *late* epoch and were dominated by domains involved in informational (red) (Table 2) and regulatory processes (green) (Figure 2A, 2E). This validated the hypothesis that informational enzymes in eukaryotes are very similar to their archaeal counterparts rather than bacterial enzymes [71]–[73]. This argument has been used to propose a sister relationship between Archaea and Eukarya and an ancient origin of Bacteria. However, our analysis revealed that sharing of informational domains between archaeal and eukaryal species was only a recent event (i.e. was evident in the *late* evolutionary epoch; *nd*≥0.55) and that the sister relationship between Archaea and Eukarya inferred from the 16S rRNA trees [74] was influenced by the high rates of modern sharing between Archaea and Eukarya (see Discussion) [75]. AE FFs were generally distributed with higher *f*-values (Figure 2E).

**Table 2:** Names, SCOP *css*, and *f*-value of informational FF domains present in the AE taxonomic group. FFs are sorted by *f*-value in a descending manner.

No.	Name	SCOP *css*	Distribution index (*f*-value)
1	L30e/L7ae ribosomal proteins	d.79.3.1	0.99
2	Ribosomal protein L3	b.43.3.2	0.99
3	L15e family	d.12.1.2	0.97
4	Ribosomal protein L10e family	d.41.4.1	0.92
5	TATA-box binding protein (TBP), C-terminal domain family	d.129.1.1	0.86
6	N-terminal domain of eukaryotic peptide chain release factor subunit 1, ERF1 family	d.91.1.1	0.80
7	DNA polymerase processivity factor	d.131.1.2	0.77
8	Sm motif of small nuclear ribonucleoproteins, SNRNP family	b.38.1.1	0.76
9	Eukaryotic DNA topoisomerase I, N-terminal DNA-binding fragment family	e.15.1.1	0.71
10	Eukaryotic DNA topoisomerase I, catalytic core family	d.163.1.2	0.71
11	eEF-1beta-like family	d.58.12.1	0.64
12	Eukaryotic type KH-domain (KH-domain type I) family	d.51.1.1	0.56
13	RNA polymerase subunit RPB10 family	a.4.11.1	0.55
14	RPB5 family	d.78.1.1	0.55
15	Ribosomal protein L19 (L19e) family	a.94.1.1	0.38
16	Ribosomal protein L13 family	c.21.1.1	0.31
17	DNA replication initiator (cdc21/cdc54) N-terminal domain family	b.40.4.11	0.27
18	Initiation factor IF2/eIF5B, domain 3 family	c.20.1.1	0.27
19	AlaX-like family	d.67.1.2	0.04
20	VMA1-derived endonuclease (VDE) PI-SceI protein	d.95.2.2	0.02

FFs unique to Archaea (A) appeared in the *late* epoch at *nd*≥0.55 and were generally distributed with lower *f*-values (Figure 2F). The discoveries of these FFs were biased towards informational and *other* domains (Figure 2A, 2F). A large number of bacteria-specific FFs (B) also appeared during the *intermediate* and *late* evolutionary epochs (Figure 2G). We note that, in general, bacterial FFs appearing in the *intermediate* epoch were biased towards informational roles while those that appeared later served metabolic and *general* roles (Figure 2A, 2G). Lastly, all of the Eukarya-specific (E) FFs appeared in the *late* epoch (Figure 2H), just like Archaea (Figure 2F). Eukarya discovered a large number of recent FF domains (*nd*≥0.55) that were involved in regulation (green circles) and extracellular processes (blue circles) and were distributed with relatively high *f*-values in the eukaryal proteomes (Figure 2A, 2H).

Superkingdom-specific FFs appeared in both Archaea and Eukarya at around the same time, and both showed a tendency to become widespread in species (Figure 2F, 2H). In contrast, the discovery of Bacteria-specific (B) FFs started much earlier but with limited spread (Figure 2G). This suggested that while Archaea was the first superkingdom to follow reductive trends, it was Bacteria that diversified first and was capable of unfolding superkingdom-specific domain structures. The primordial stem-line (that was structurally and functionally complex) later evolved into eukaryotes, possibly after engulfment of already diversified microbes (Discussion). In this regard, we identified a set of mitochondrial FFs, all of which appeared at *nd*≥0.55, during and after the rise of the E taxonomic group, including the ‘Mitochondrial resolvase ydc2 catalytic domain’ (c.55.3.7; *nd* = 0.55) and the ‘Mitochondrial cytochrome c oxidase subunit VIIb’ (f.23.5.1; *nd* = 0.59) FFs (Table 3). Thus, our timelines do not support fusion hypotheses for the origin of eukaryotes linked to a confluence between akaryotes. The fusion scenarios have been discussed elsewhere [65], [70], [76]–[79] and it is beyond the scope of this study to evaluate what model is better. In light of our data that is based on the genomic census of conserved FF domains in hundreds of free-living organisms, we support a phagotrophic and eukaryote-like nature of the host (anticipated in [78], [79]) that acquired the primordial alpha-proteobacterium as an endosymbiont, which later became mitochondria and triggered the diversification of eukaryotes (at *nd* = 0.55; roughly ∼1.6 billion years ago). A formal test of this hypothesis is warranted and will be explored in a future study. The exercise also revealed that the lower median *f*-values observed earlier (Figure 1C) were due to the significant drop in *f* in the *intermediate* evolutionary epoch. We note that the majority of the bacterial FFs (belonging to the ABE, BE, B and AB taxonomic groups) also appeared during this period and thus affected the overall medians.

**Table 3:** Names, SCOP Id and *css*, and evolutionary age (*nd*) of FFs that were identified by keyword search ‘Mitochondria’ on the dataset of 2,397 FF domains.

SCOP Id	SCOP *css*	Description	Age (*nd*)
69533	c.55.3.7	Mitochondrial resolvase ydc2 catalytic domain	0.55
81422	f.23.5.1	Mitochondrial cytochrome c oxidase subunit VIIb	0.59
81426	f.23.6.1	Mitochondrial cytochrome c oxidase subunit VIIc (aka VIIIa)	0.59
81418	f.23.4.1	Mitochondrial cytochrome c oxidase subunit VIIa	0.63
111358	f.45.1.1	Mitochondrial ATP synthase coupling factor 6	0.64
81414	f.23.3.1	Mitochondrial cytochrome c oxidase subunit Vic	0.65
54530	d.25.1.1	Mitochondrial glycoprotein MAM33-like	0.71
81410	f.23.2.1	Mitochondrial cytochrome c oxidase subunit Via	0.71
81405	f.23.1.1	Mitochondrial cytochrome c oxidase subunit IV	0.73
47158	a.23.4.1	Mitochondrial import receptor subunit Tom20	0.74
103507	f.42.1.1	Mitochondrial carrier	0.96

FFs are sorted by *nd* value in an ascending manner.

### Phylogenomic patterns

**Figure 3:**
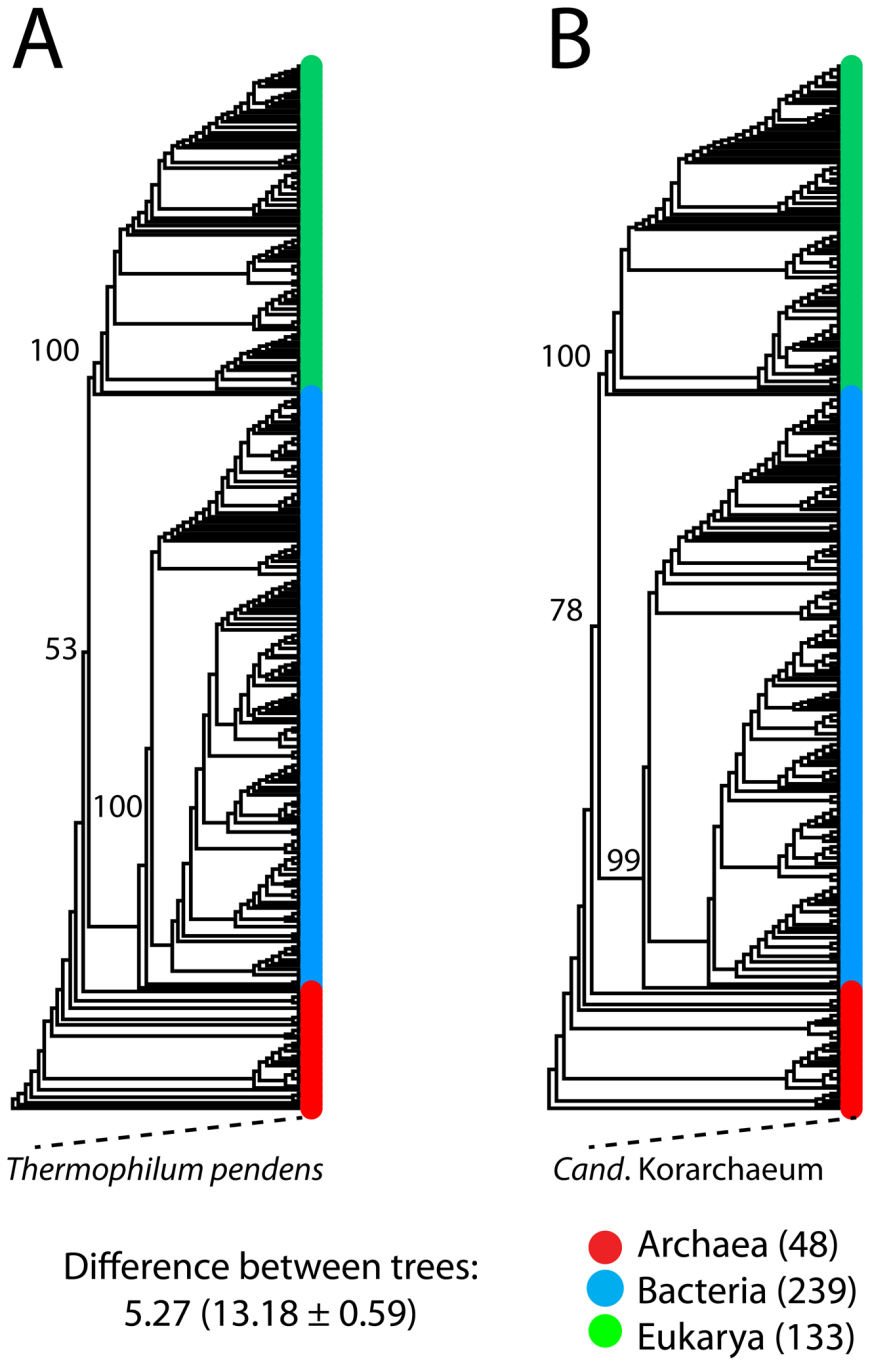
Phylogenomic patterns in the three superkingdoms. A) A ToL reconstructed from the genomic abundance counts of 2,397 FF domains (2,262 parsimony informative, tree length = 128,752, RI = 0.76, *g_1_* = −0.33) describing the evolution of 420 free-living organisms. Values on branches indicate bootstrap support values. Taxa were colored red for Archaea, blue for Bacteria and green for Eukarya. B) A ToL reconstructed from the presence/absence of 2,397 FF domains (2,249 parsimony informative, tree length = 30,599, RI = 0.79, *g_1_* = −0.28) describing the evolution of 420 free-living organisms. Values on branches indicate bootstrap support values. Taxa are colored as in A. Difference between trees was calculated using the nodal module of TOPD/FMTS package [50].

We generated rooted ToLs from *abundance* (Figure 3A) and *occurrence* (Figure 3B) counts of 2,397 FF domains in the 420 free-living proteomes (see Dataset S1 for taxon names) using maximum parsimony as the optimality criterion in PAUP* 4.0b10 [44]. Both reconstructions recovered a previously established tripartite world of cellular organisms [20], [27], [74], [80]. The archaeal superkingdom always formed a paraphyletic group at the base of the ToLs. The deep branches of the ToLs were occupied by thermophilic and hyperthermophilic archaeal species (*Thermofilum pendens* and *Cand*. Korarchaeum) (Figure 3). The archaeal rooting of the ToL is supported by a number of previous studies (e.g. [14], [20], [27], [81]–[83]) and is in conflict with the traditional Archaea-Eukarya sister relationship (Discussion). Bacteria and Eukarya formed strong monophyletic clades that were supported by high BS values (≥99%) and were separated from Archaea with 53% (Figure 3A) and 78% (Figure 3B) BS support. Both ToLs had strong phylogenetic signal (*g*_1_ = −0.33 and −0.28). Overall, phylogenomic patterns resembled traditional groupings and supported previous analyses of similar kind [20], [27]. Moreover, the dissimilarity between two reconstructions was 5.37, which was smaller than the mean RMSD calculated from 100 random comparisons (Figure 3) (Methods). Because the ToLs were supported with high confidence and resembled previous analyses [20], [27], they made useful tools for the study of domain gain and loss events on the many branches (read below).

### Global patterns of domain gains and losses

To quantify the relative contributions of domain gains and losses impacting the evolution of superkingdoms, we retraced the history of character state changes (i.e. changes in the abundance or occurrence of FFs) on each branch of the reconstructed ToLs. For each FF domain, we counted the number of times it was gained and lost in different branches of the phylogenetic tree. Gains were recorded when the abundance/occurrence of a particular FF at a node was higher than the corresponding value at the immediate ancestral node. In contrast, losses were incremented when the abundance/occurrence of a particular FF at a node was lower. Because we allowed character changes in both forward and backward directions (Wagner parsimony), each FF character could be both gained and lost a number of times across the many branches of the ToL. This assumption is reasonable as different lineages of organisms utilize domain repertoires differently. Because abundance counts are expected to be higher in the eukaryotic species (especially in metazoa) due to increased gene duplication events and a persistence strategy that favors flexibility and robustness (Figure 1D) [64], we also considered gains and loss statistics from the *occurrence* trees.

To evaluate the performance of both models, we first compared the number of FFs that were gained (i.e. net sum above zero) and lost (net sum below zero) in both reconstructions. Out of the total 2,397 (2,262 parsimony informative) FF domains in the *abundance* model, 1,955 (86%) were gained, while only 236 (10%) were lost (Dataset S2). In contrast, *occurrence* identified 60.1% FFs as gained (1,353/2,249) and 30.5% (686/2,249) as lost (Dataset S3). Nearly 96% (1300/1,353) of the *occurrence* gains were also gained in *abundance* while only 26% (178/686) losses were common to both models. This suggested that *abundance* included nearly all the *occurrence* gains and likely overestimated the number of gains (due to gene duplications and domain reuse). In contrast, *occurrence* led to more balanced distributions and likely overestimated losses (read below).

**Figure 4:**
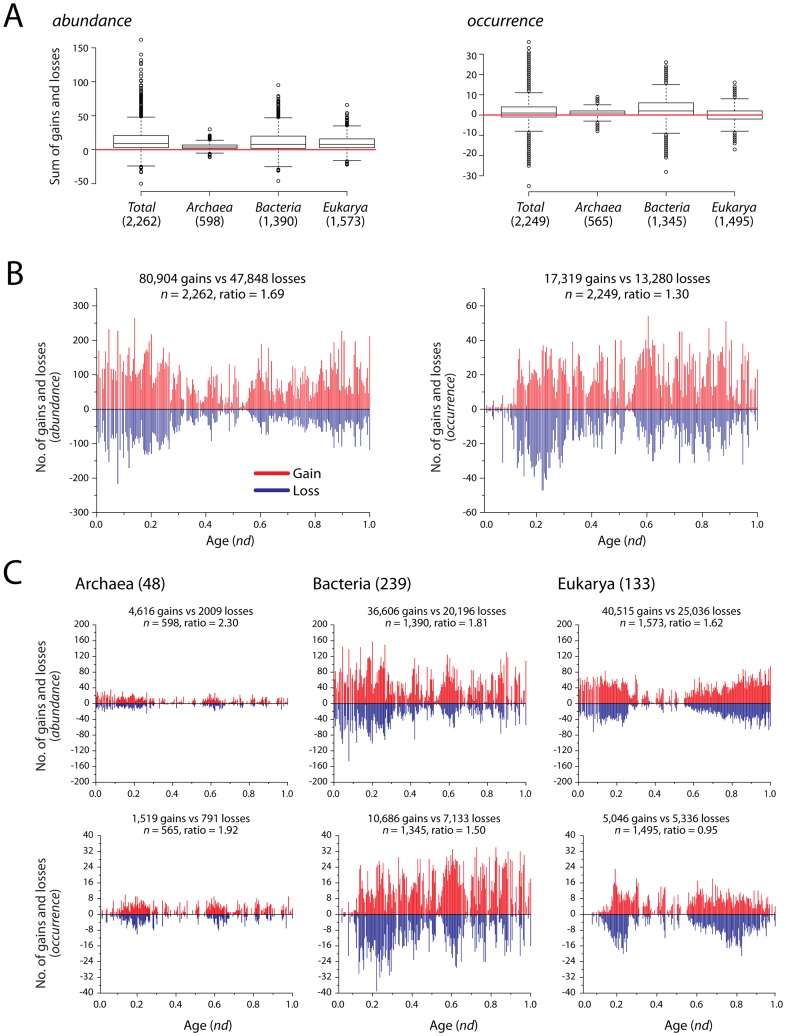
Global patterns of gains and losses in superkingdoms. A) Sum of gains and losses for each FF domain is represented in boxplots for *Total*, *Archaea*, *Bacteria*, and *Eukarya* reconstructions using *abundance* and *occurrence* models. Numbers in parentheses indicate total number of parsimony informative characters in each analysis. A horizontal red line passes through zero on the *x*-axis. B) Histograms comparing the relative counts of gains and losses for each FF domain character, plotted on the *nd* scale. Bars in red and blue indicate gains and losses respectively. The global gain-to-loss ratios are listed along with the total number of gain and loss events and gain-to-loss ratios. *n* is the number of parsimony informative characters in each analysis. C) Histograms comparing the distribution of FF gains and losses in *Archaea*, *Bacteria* and *Eukarya*. Bars in red and blue indicate gains and losses respectively. The *x*-axes indicates evolutionary time. Numbers in parenthesis indicate total number of proteomes in each dataset.

To provide additional support to the gain/loss model, we pruned taxa from the original ToLs leaving only one superkingdom and recalculated character state changes on the pruned trees. This eliminated any biases resulting from the differences in the persistence strategies of the three superkingdoms and yielded four phylogenetic trees, *Total* (taxa = 420, total FF characters = 2,397), *Archaea* (48, 703), *Bacteria* (239, 1,510) and *Eukarya* (133, 1,696). For each of the four trees, we calculated the sum of gain and loss events for all parsimony informative FF characters and represented the values in boxplots (Figure 4A). In all distributions, medians were above 0 indicating that the sum of net gains and losses was a non-negative number for both *abundance* (Figure 4A:*abundance*) and *occurrence* (Figure 4A:*occurrence*) models. The exception was the eukaryal tree pruned from the *occurrence* model, for which the median was exactly zero. The result revealed that while both gains and losses occurred quite frequently, the former was more prevalent in proteome evolution.

The histograms in Figure 4B describe the distributions of gain and loss counts for all parsimony informative FF characters in the *Total* dataset. When plotted against evolutionary time (*nd*), results highlighted remarkable patterns in the evolution of domain repertoires. Domain gains outnumbered losses in both *abundance* (80,904 gains vs. 47,848 losses) and *occurrence* (17,319 vs. 13,280) tree reconstructions (Figure 4B). The gain-to-loss ratios were 1.69 and 1.30, respectively, indicating an increase of 69% and 30% in gains relative to losses. Relative differences in the numbers of gains (red) versus losses (blue) suggested that gains increased with the progression of evolutionary time in both reconstructions (read below).

We note that different evolutionary processes may be responsible for shaping the proteomes in individual superkingdoms. For example, the origin of Archaea has been linked to genome reduction events [20], [84], while HGT is believed to have played an important role in the evolution of bacterial species [25]. In contrast, eukaryal proteomes harbor an increased number of novel domain architectures that are a result of gene duplication and rearrangement events [6], [43]. Therefore, to eliminate any biases resulting from the effects of superkingdoms in the global analysis (Figure 4B), we recalculated the history of character changes on the pruned superkingdom tress recovered earlier (Figure 4C). For *abundance* reconstructions, the exercise supported earlier results where the number of gains was significantly higher than the corresponding number of losses for Archaea (4,616 vs. 2,009), Bacteria (36,606 vs. 20,196), and Eukarya (40,515 vs. 25,036) (Figure 4C: *abundance*). The overall gain to loss ratios decreased from 2.30 in Archaea to 1.81 in Bacteria and 1.62 in Eukarya (Figure 4C: *abundance*). The increased gain-to-loss ratios in akaryotic microbial species are remarkable; it implies that the rate of gene discovery in akaryotic microbes (by *de novo* creation, gene duplication, acquisition by HGT and/or recruitment) is higher than the rate in eukaryotes. This tendency in microbial species could be a novel ‘collective’ persistence strategy to compensate for their economical proteomes. For histograms representing *occurrence* models, global gain-to-loss ratios decreased in the order, Archaea>Bacteria>Eukarya (Figure 4C: *occurrence*). Remarkably, the ratio in Eukarya dropped below 1 indicating prevalence of domain loss events relative to gains. This result supports recent studies that have proposed the evolution of newly emerging eukaryal phyla via genome reduction [85].

### Accumulation of gains and losses in evolutionary time

**Figure 5:**
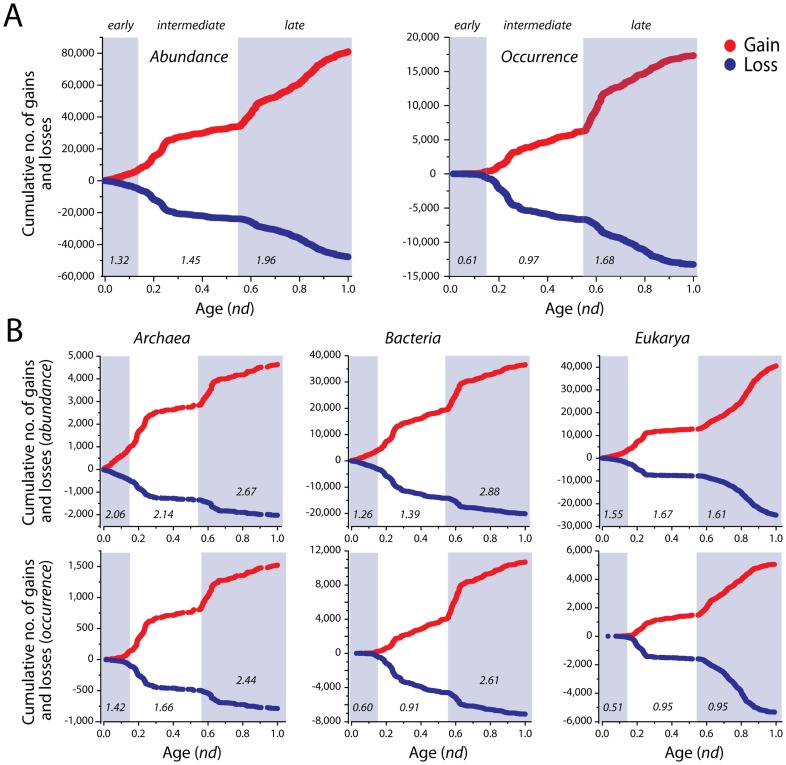
Cumulative numbers of gains and losses. Scatter plots reveal an approximately linear trend in the accumulation of FF gains and losses in both the global analysis (A) and in individual superkingdoms (B). Gains are identified in red while losses in blue. The three evolutionary epochs are marked with corresponding gain-to-loss ratios in italics.

When partitioned into the *early*, *intermediate*, and *late* evolutionary epochs, the gain-to-loss ratios exhibited an approximately linear trend towards increasing gains (Figure 5). For *abundance*, the ratios increased from 1.32 in the *early* epoch to 1.45 in the *intermediate* and 1.96 in the *late* evolutionary epochs. Similar trends were also observed for *occurrence*, with calculated ratios of 0.61, 0.97, and 1.68, respectively (Figure 5A). In fact, both gains and losses increased linearly with evolutionary time in all reconstructions. However, accumulation of gains overshadowed the number of losses (Figure 5). Remarkably, the *occurrence* model suggested predominant losses in the first two phases of evolution (0.61 and 0.97) that were compensated by significantly higher amounts of gains (1.68) in the *late* epoch. In contrast, *abundance* failed to illustrate this effect and indicated overwhelming gains in all evolutionary epochs.

When looking at the individual epochs for pruned trees (Figure 5B), we noticed that the rate of domain gain increased with time (as before) (Figure 5A). However, the ratios in the initial two evolutionary epochs were considerably higher in Archaea for both the *abundance* and *occurrence* models. For example, Archaea exhibited gain-to-loss ratios of 2.06 and 2.14, in comparison to 1.26 and 1.39 in Bacteria, and 1.55 and 1.67 in Eukarya for *early* and *intermediate* evolutionary epochs (Figure 5B:*abundance*). In contrast, Bacteria exhibited an overwhelming gain-to-loss ratio of 2.88 in comparison to 2.67 in Archaea and 1.61 in Eukarya, in the *late* evolutionary epoch. Overall, the gain-to-loss ratios increased with evolutionary time in all superkingdoms with the sole exception of Eukarya that had a lower ratio in the *late* (1.61) compared to the *intermediate* (1.67) epoch (Figure 5B:*abundance*).

Results based on *occurrence* indicated similar trends but with relatively more balanced gain-to-loss ratios and still highlighted the abundance of domain gains in evolution. The individual ratios were 1.42, 1.66, and 2.44 in Archaea, 0.60, 0.91, and 2.61 in Bacteria, and 0.51, 0.95, and 0.95 in Eukarya (Figure 5B:*occurrence*). Both Bacteria and Eukarya showed increased levels of ancient domain loss. However, Bacteria compensated this decrease by engaging in massive gain events during the *late* evolutionary epoch (ratio of 2.61). In contrast, Eukarya exhibited an even exchange between FF gain and loss events (ratio = 0.95) in both the *intermediate* and *late* epochs. *Occurrence* results also supported the evolution of Eukarya by gene loss, which is in line with recently published analyses [23], [85]. *Abundance* also indicated this drop in gene discovery rate for recent domains in Eukarya. However, the drop appears to be compensated by increased duplications of other domains that lead to an increase in the overall number of domains that are gained (Figure 5B: *abundance*). This apparent discrepancy can be explained by the power of both models in depicting true evolutionary relationships between organisms. *Abundance* accounts for a number of evolutionary processes such as HGT, gene duplication, and gene rearrangements while *occurrence* merely describes presence and absence of FFs and because of its more ‘global’ nature fails to illustrate a complete evolutionary picture (Discussion).

### Effect of unequal sampling of proteomes

**Figure 6:**
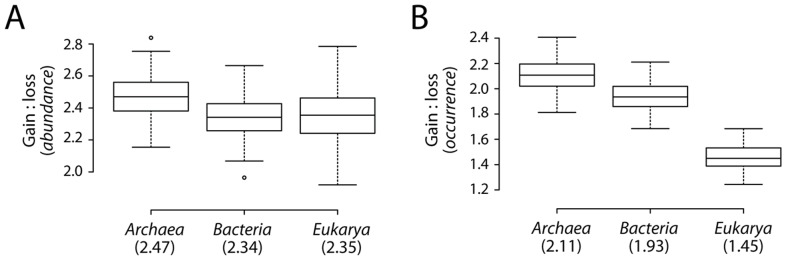
Equal sampling of proteomes. Boxplots comparing the distribution of net gains and losses in 100 random phylogenetic trees for both *abundance* (A) and *occurrence* (B). Numbers in parentheses indicate group median values.

To test whether unequal sampling of proteomes per superkingdom was contributing any bias to the calculations of domain gains and losses, we extracted 100 random samples of 34 proteomes each from the three superkingdoms and generated 100 random trees. From each of the random trees, we recalculated the gain-to-loss ratios using both *abundance* and *occurrence* models (Figure 6). Random and equal sampling supported the overall conclusion that gains were overwhelming during the evolution of domain repertoires (Figure 6). The median ratios for random trees were 2.47 in Archaea, 2.35 in Eukarya, and 2.34 in Bacteria for *abundance* reconstructions (Figure 6A). In comparison, the ratios decreased from 2.11 in Archaea to 1.93 in Bacteria and 1.11 in Eukarya for *occurrence* reconstructions (Figure 6B). Based on the results of random and equal sampling, we safely conclude that the gain of domains in proteomes is a universal process that occurs in all three superkingdoms of life. Moreover, the gain-to-loss ratios increase with time (Figure 5) and their effects are directly responsible for evolutionary adaptations in superkingdoms (Discussion). We also propose that using *abundance* increases the reliability of the phylogenomic model and accounts for many important evolutionary events, a feat that is not possible when studying *occurrence*.

### GO enrichment analysis

We identified FFs that were gained (i.e. net sum of gains and losses was above 0) and lost (net sum below 0) directly from the pruned superkingdom trees. To eliminate any redundancy, we only kept FFs that were gained (or lost) in both *abundance* and *occurrence* reconstructions and excluded those where both methods disagreed. Using this stringent criterion, we classified a total of 368 archaeal FFs as being gained and 40 as being lost. In comparison, Bacteria and Eukarya gained 892 and 633 FFs, respectively, while they lost only 148 and 164 FFs. Both gained and lost FFs for each superkingdom were provided as input to the online dcGO resource [56], [57] to retrieve the highly specific and significantly enriched biological process GO terms (Methods). For FFs that were gained, a total of six GO terms were significantly enriched in archaeal proteomes representing biological processes involved in the biosynthesis of nucleotides and metabolism, such as ‘tricarboxylic acid cycle [GO:0006099]’, ‘pyruvate metabolic process [GO:0006090]’, ‘acyl-CoA metabolic process [GO:0006637]’, ‘thioester biosynthetic process [GO:0035384]’, ‘purine nucleobase metabolic process [GO:0006144]’, and ‘pyrimidine nucleoside metabolic process [GO:0006213]’ (Table 4). In comparison, only one biological process in Bacteria (‘polysaccharide catabolic process [GO:0000272]’) and 37 in Eukarya were significantly enriched (Table 4). While, the bacterial GO term corresponded to metabolic roles (similar to Archaea), eukaryal functions encompassed a diverse range of processes including ‘sex determination [GO:0007530]’, regulatory [GO:0044089] and immunological roles [GO:0046634], functions related to the development of mammary glands [GO:0061180], and others (Table 4). Finally, none of the archaeal or eukaryal lost FFs was significantly associated with any of the highly-specific biological process GO terms, indicating that loss of FFs in these two superkingdoms occurred without any functional constraint. In contrast, two biological processes were predicted to be lost from Bacteria including, ‘cellular modified amino acid biosynthetic process [GO:0042398]’, and ‘pyrimidine-containing compound biosynthetic process [GO:0072528]’(Table 5).

**Table 4:** GO accessions, names and *P*-values for highly-specific biological processes that were significantly associated (FDR<0.01) with FF gains in Archaea, Bacteria, and Eukarya.

Superkingdom	No.	GO accession	Biological processes	*P*-value
Archaea	1	GO:0006099	tricarboxylic acid cycle	5.38E-06
	2	GO:0006090	pyruvate metabolic process	2.80E-05
	3	GO:0006637	acyl-CoA metabolic process	4.01E-05
	4	GO:0035384	thioester biosynthetic process	3.32E-04
	5	GO:0006144	purine nucleobase metabolic process	5.71E-04
	6	GO:0006213	pyrimidine nucleoside metabolic process	6.38E-04
Bacteria	1	GO:0000272	polysaccharide catabolic process	1.26E-04
Eukarya	1	GO:0045995	regulation of embryonic development	1.44E-06
	2	GO:0051588	regulation of neurotransmitter transport	3.35E-06
	3	GO:0001707	mesoderm formation	7.48E-06
	4	GO:0001649	osteoblast differentiation	1.29E-05
	5	GO:0050870	positive regulation of T cell activation	3.45E-05
	6	GO:0030336	negative regulation of cell migration	8.88E-05
	7	GO:0048017	inositol lipid-mediated signaling	1.05E-04
	8	GO:0000165	MAPK cascade	1.16E-04
	9	GO:0051291	protein heterooligomerization	1.21E-04
	10	GO:0046620	regulation of organ growth	2.43E-04
	11	GO:0051099	positive regulation of binding	3.00E-04
	12	GO:0043627	response to estrogen stimulus	3.00E-04
	13	GO:0051216	cartilage development	2.96E-04
	14	GO:0061180	mammary gland epithelium development	2.96E-04
	15	GO:0030856	regulation of epithelial cell differentiation	3.02E-04
	16	GO:0051703	intraspecies interaction between organisms	4.13E-04
	17	GO:0032496	response to lipopolysaccharide	4.07E-04
	18	GO:0032946	positive regulation of mononuclear cell proliferation	5.10E-04
	19	GO:0032869	cellular response to insulin stimulus	5.10E-04
	20	GO:0045580	regulation of T cell differentiation	6.59E-04
	21	GO:0060191	regulation of lipase activity	6.59E-04
	22	GO:0045834	positive regulation of lipid metabolic process	6.59E-04
	23	GO:0050673	epithelial cell proliferation	6.59E-04
	24	GO:0021761	limbic system development	8.39E-04
	25	GO:0046634	regulation of alpha-beta T cell activation	8.39E-04
	26	GO:0045667	regulation of osteoblast differentiation	8.39E-04
	27	GO:0007492	endoderm development	8.39E-04
	28	GO:0044089	positive regulation of cellular component biogenesis	1.04E-03
	29	GO:0007530	sex determination	1.04E-03
	30	GO:0045598	regulation of fat cell differentiation	1.04E-03
	31	GO:0051057	positive regulation of small GTPase mediated signal transduction	1.25E-03
	32	GO:0048749	compound eye development	1.31E-03
	33	GO:0050773	regulation of dendrite development	1.31E-03
	34	GO:0060443	mammary gland morphogenesis	1.31E-03
	35	GO:2001236	regulation of extrinsic apoptotic signaling pathway	1.31E-03
	36	GO:0016055	Wnt receptor signaling pathway	1.31E-03
	37	GO:0046488	phosphatidylinositol metabolic process	1.31E-03

**Table 5:** GO accessions, names and *P*-values for highly-specific biological processes that were significantly associated (FDR<0.01) with FF loss in Bacteria.

Superkingdom	No.	GO accession	Biological processes	*P*-value
Bacteria	1	GO:0042398	cellular modified amino acid biosynthetic process	3.10E-04
	2	GO:0072528	pyrimidine-containing compound biosynthetic process	3.10E-04

No significant biological process was lost in either Archaea or Eukarya.

## Discussion

### Evolutionary patterns

We report the evolutionary dynamics of gain and loss events of protein domain FFs in hundreds of free-living organisms belonging to the three cellular superkingdoms. Structural phylogenomic methods were used to reconstruct ToLs from genomic *abundance* and *occurrence* of FF domains in proteomes. Standard character reconstruction techniques were then used to trace domain gain and loss events along the branches of the universal trees. Finally, molecular functions and biological processes of FFs were studied using traditional resources. The exercise revealed remarkable patterns:

#### (1) Domain gains outnumbered losses throughout evolution

The tracing of character state changes along the branches of ToLs revealed that both domain gain and loss were frequent outcomes in proteome evolution. However, a global trend of gains was pervasive along the entire evolutionary timeline and in all superkingdoms (Figures 4 and 6). Remarkably, the gain-to-loss ratios increased with the progression of evolutionary time (Figure 5). However, the rates of domain discovery varied considerably among superkingdoms. Domain gain can lead to interesting evolutionary outcomes. First, it increases the domain repertoire of cells and enhances the persistence strategies of living organisms. Second, the process allows acquisition of novel functions and ensures the availability of more domains for use in the combinatorial interplay that is responsible for the generation of novel domain architectures. In contrast, gene loss events are important for changes from free-living to parasitic or symbiotic lifestyles (e.g. [21], [22]) that lead to highly reduced genomes. To our knowledge, this is the first exercise that has studied gain-and-loss dynamics on a global scale by subjecting all organismal lineages in a ToL to character state reconstruction analysis.

#### (2) Secondary evolutionary adaptations are ongoing in superkingdoms

Modeling of FF gain and loss events in proteomes revealed that microbial superkingdoms, especially Archaea, had the highest rates of domain gains (Figures 4 and 5). This finding and the fact that the majority of the informational FFs unique to the AE taxonomic group (Table 2) were late additions (*nd*≥0.55) to the FF repertoires point to another interesting evolutionary adaptation of Archaea: the late discovery and sharing of FFs with other superkingdoms (especially Eukarya) to compensate for the initial evolutionary reductive trend. This secondary archaeal adaptation to offset ancient genome reduction events and the proteomic trends towards economy may also be occurring (albeit at lower degree) in Bacteria, which also exhibited higher levels of gene discovery. In contrast, eukaryal species favored the reuse of already existing domains rather than engaging exclusively in novel domain discovery. Thus, akaryotic microbes persist by fostering trends towards economy while eukaryotic species favor patterns of more flexibility and robustness (Figure 1D). However, the low robustness of archaeal proteomes is intriguing and demands an explanation. Archaea are characterized by their preferences for extreme environmental niches (e.g. thermophilic and halophilic environments), a factor intuitively responsible for increased robustness in cells. However, robustness is associated with an organismal ability to respond to changing environmental conditions [64]. Both Bacteria and Eukarya are more diverse in this regard and interact with a diverse range of temperatures, moistures, and climates. In comparison, Archaea are more restricted in terms of their environmental niches and do not generally face varied climatic conditions. In light of these observations, our finding that robustness in cells increased in the order, Archaea, Bacteria, and Eukarya is intuitively well supported.

#### (3) Functional annotations of timelines revealed differential enrichment of molecular functions in superkingdoms

Annotations of the molecular functions of FFs highlighted the abundance of metabolic and informational domains in proteomes (Figure 2A), supporting previous studies [21]. Informational FFs were significantly over-represented in the AE taxonomic group and appeared during the *late* evolutionary epoch. This suggested that both Archaea and Eukarya work with a very similar apparatus for decoding their genetic information, which is different from Bacteria. However, as we explained above, all these innovations occurred in the *late* epoch (*nd*>0.55), highlighting ongoing secondary adaptations in the superkingdoms. In comparison, the BE taxonomic group was enriched in metabolic FFs (Figure 2A). This toolkit was probably acquired via HGT during endosymbiosis of primordial microbes rich in diverse metabolic functions (read below).

The enrichment of biological processes in superkingdoms revealed that akaryotes gained and lost metabolic capabilities during the course of evolution (Tables 4 and 5), while eukaryotes gained a significant number of functionalities involved in the diversification of eukaryal lineages such as the development of mammary glands, compound eye development, enhanced regulatory roles, and sex determination (Table 4). All these processes reflect relatively recent evolutionary innovations in the eukaryal superkingdom suggesting that while the overall rate of innovation was lowest in Eukarya; it was directed towards discovering important functions responsible for the diversification of eukaryal phyla and kingdoms (e.g. appearance of mammals) from the last common eukaryotic ancestor. However, we caution that the significantly enriched GO terms (Tables 4 and 5) only represent a subset of FFs (i.e. those corresponding to gains and losses) from the entire FF repertoires in superkingdoms. Thus they do not reflect the entire toolkit of biological processes that are expected to occur in the living organisms and should be interpreted with limited scope.

#### (4) Early origin of Archaea by genomic streamlining

ToLs generated from genomic *abundance* and *occurrence* counts were rooted paraphyletically in Archaea, a result that disagrees with the canonical rooting of Bacteria recovered from 16S rRNA and ancient paralogous gene sequence trees [74], [86]. The archaeal rooting of the universal tree is supported by a number of previous studies involving more conserved phylogenetic characters describing the structure and function of both proteins and RNA molecules [38], [82], [83], [87], [88]. We have previously argued that trees built from protein domain structure (i.e. FSFs and FFs) are robust against a number of problems that complicate phylogenetic analysis of gene sequences [37]. First, gene sequences are prone to high mutation rates [89] and are far less conserved than protein domain structures [20]. Second, computation of a reliable sequence alignment is a painstaking process and often involves manual editing [90]. Third, alignment forces unnecessary assumptions about inapplicable characters such as insertion/deletions [91], [92]. Fourth, sequence sites in genes interact with each other to form secondary structures and domain regions and consequently do not change independently from each other [93]–[95]. Thus each nucleotide cannot be considered an independent character in phylogenetic analyses [37]. These and other shortcomings (see [37]) limit and reduce the reliability of sequence-based methods and cast doubt on statements of deep phylogeny such as the canonical rooting of the ToL. Moreover, the 16S rRNA gene that is considered the gold standard for phylogenetic analysis only represents one component of the ribosome, a central macromolecular complex that holds at least two other rRNA components and many structural proteins with varying evolutionary histories [96]. Thus, trees built from rRNA genes can only provide a glimpse of the evolutionary history of the ribosome and not the entire organismal systems that are made up of many biological parts. Our approach is advantageous in this regard as it studies the evolution of systems (organisms) using their component parts (entire domain repertoire) and provides a global perspective. Finally, our approach does not require computation of any alignment and does not violate the assumption of character independence, as each SCOP FF is an independent evolutionary unit [37].

The distribution of FF domains in superkingdoms also showed that both the numbers of unique and shared FFs were lowest in Archaea. For example, the number of FFs shared between Bacteria and Eukarya was considerably higher than those shared with Archaea (BE = 412 vs. AB = 90 and AE = 40) (Figure 1A). Without any formal phylogenetic analysis, it is evident from the patterns of use and sharing of domain structures in Venn diagrams (Figure 1A) and the 3D-plots describing persistence strategies (Figure 1D), that Archaea represents the simplest form of cellular life. The smaller FF domain repertoires in archaeal species could be an outcome of one of two possible events: (i) Archaea evolved by gradual loss of ancestral genes via genome reduction when nascent lineages delimited the emergence of the first superkingdom of life, or (ii) Both Bacteria and Eukarya gained a significant number of FFs later in evolution after diverging from Archaea, while the archaeal superkingdom persisted in its path of economy. While both of these scenarios point to an early origin of the archaeal superkingdom, our data and previous results [27] are more compatible with the former event.

We have previously argued that the complete absence of an ‘ancient’ fold in one superkingdom more likely represents a loss event in that superkingdom rather than simultaneous gains of the same fold in other superkingdoms (e.g. [20]). In other words, the probability of one group loosing a structure is higher than two groups acquiring the same structure at the same time. Under this probabilistic model, the appearance of the BE taxonomic group at *nd* = 0.15 represents a fundamental evolutionary event of complete loss of ancient FFs in the archaeal superkingdom (Figure 1B). Our data confirm that the first FF to be lost from Archaea was the ‘Heat-shock protein, HSP90, N-terminal domain’, which is highly conserved in bacterial and eukaryotic species but completely absent in Archaea. Lack of HSP90 chaperones in Archaea is intriguing and merits future exploration of how protein-folding mechanisms work in extremophiles. A recent analysis of FSF domains [14] also confirmed that Archaea evolved by genome reduction and that this process started very early in evolution. In that study, the distribution (*f*-value) of 1,739 FSFs in 70 archaeal proteomes revealed that many of the ancient folds were completely absent in archaeal species. This hypothesis is strengthened by our data of minimal sharing of FFs in archaeal taxonomic groups (Figure 1A) and the appearance of taxonomic groups (Figure 1B), suggesting an early evolutionary split of Archaea (Figure 3). In light of these observations, our finding that the origin of diversified cellular life lies in thermophilic archaeal species (Figure 3) is a significant outcome that is supported by sound methodological and evolutionary considerations.

#### (5) A canonical pattern of superkingdom diversification embeds the likely endosymbiotic origin of eukaryotes

FF distributions in the evolutionary timeline of domain appearance revealed that Archaea was the first superkingdom to materialize by selective loss of domain structures at the end of the *early* epoch of evolution (Figure 1B and 2). Remarkably, however, the appearance of superkingdom-specific domains followed an order that matches the canonical pattern of early rise of Bacteria during the *intermediate* epoch and joint rise of diversified Archaea and Eukarya at the start of the *late* epoch. Thus, the primordial stem line, which was already structurally and functionally quite complex, generated organismal biodiversity first by streamlining the structural make up in Archaea (at *nd* = 0.15), then by generating novelty in Bacteria (*nd* = 0.26), and finally by generating novelty and co-opting bacterial lineages as organelles in Eukarya (*nd*<0.55). The eukaryotic group was able to deploy massive structural and functional innovation despite concomitant streamlining, which we show spread through eukaryotic lineages at high frequency (Figure 2). Tendencies of flexibility and robustness of this kind were neither deployed by the akaryotic superkingdoms that preceded Eukarya nor by superkingdom-specific diversification of the archaeal domain repertoires that coincided with its rise.

Our data is incompatible with fusion scenarios between akaryotic cells that are used to explain the origin of eukaryotes [66]–[68], [97], which have been criticized previously [70], [76]–[79] and are not supported by comparative proteomics analysis [78]. They also fail to explain the presence of bacterial-like lipids in eukaryotes, especially if the partner cells were of archaeal and bacterial origin (e.g. [67]). Moreover, no known mechanism of akaryotic engulfment exists, no extant bacterium is known to enter or survive inside archaeal organisms, and cellular fusion is incompatible with archaeal cell biology. In contrast, there is considerable evidence supporting the endosymbiotic origins of eukaryotic organelles. It is highly likely that mitochondria developed from the SAR11 clade of marine bacteria, a sister group to the Ricketsialles [98]. There is also considerable evidence in support of eukaryotic mechanisms of phagocytosis that would enable microbial engulfment of organelle ancestors [99]. The question however relates to the defining event of eukaryal diversification. Our timelines indicate the presence of an ancestral proto-eukaryotic stem lineage that was structurally and metabolically quite advanced. This lineage already produced superkingdoms Archaea and Bacteria by genomic streamlining, which was likely triggered by a host of selective pressures, including the escape from viruses and phagotrophs, the need to adapt to extreme environments (Archaea), and the benefits of rapid growth (Bacteria) [70]. The early rise of diversified Bacteria thus supports the existence of alpha-proteobacterial ancestors of mitochondria before the appearance of diversified eukaryotes 1.6 Gy ago (*nd* = 0.55), as indicated by microfossil evidence and the molecular clock [51], [52]. The fact that the first mitochondrial-specific FFs appeared at that time (Table 3) boosts the idea of the joint rise of Eukarya and eukaryotic organelles. It is therefore highly likely that the proto-eukaryotic stem line acquired phagotrophic abilities and engulfed an alpha-proteobacterium and other microbes (including microbes of archaeal origin) to trigger the diversification of eukaryotes soon after. This scenario seems most compatible with our timelines and explains the enrichment of metabolic BE domains. A formal test of the phagotrophic proto-eukaryotic ancestor is warranted.

### Reliability of our study

How reliable is our study? Both *abundance* and *occurrence* were congruent with respect to the overall tree topologies and general conclusions drawn from the analyses. Both supported the existence of overwhelming gains in evolution. However, discrepancies also existed especially in the numerical differences for the gain-to-loss ratios among superkingdoms. In general, *abundance* (apparently) overestimated gains while *occurrence* underestimated losses. The higher number of gain-to-loss ratios in *abundance* models is an expected outcome as we are accounting for evolutionary processes such as gene duplications, gene rearrangements, and HGT that are known to increase the representation of genes in genomes. Ancient genes have more time to multiply and increase their genomic abundance compared to newly emergent genes. In contrast, *occurrence* merely describes the presence or absence of genes and provides a simplified view of the overall landscape of change. Another explanation is the possible existence of methodological artifacts when dealing with genomic occurrence in parsimony analysis that excludes most of the ancient FFs as non-informative characters, when these are present in all proteomes. Moreover, *occurrence* fails to take into account the weighted contribution of ancient genes to the phylogeny and treats all characters equally. Thus trees built from *abundance* counts are better resolved at their base while trees built from *occurrence* behave poorly in this regard [27]. We emphasize that the focus of this study is to highlight the relative contribution of domain gains and losses in the evolution of superkingdoms and not to evaluate which methodology is preferable. The finding that domain gains are overwhelming and increase approximately linearly with evolutionary time in both models is remarkable and suggests that the appearance of novel domains is a continuous process (Figures 4 and 5).

In our phylogenomic model, we rooted ToLs by character absence (i.e. 0) using the Lundberg method. We assumed that proteomes became progressively richer during the course of evolution. However, this implicit assumption did not lead to an increased number of domain gains as character state changes in both forward (e.g. 9 to 22) and reverse (12 to 5) directions were allowed and carried equal weights. Moreover, we evaluated the effects of ToL rooting on the calculations of domain gain and loss statistics by considering outgroup taxa instead of the Lundberg method. Superkingdom trees rooted with outgroup taxa led to similar tree topologies and supported the conclusion of overwhelming gains that we here report (Figure S1). However, we decided to exclude outgroup analysis from this study for two reasons. First, outgroups add an external hypothesis into the model and bias gains and losses by including artificial character changes in the most basal branches leading to outgroup taxa. Second, the selection of the most appropriate outgroups for each superkingdom is a complicated problem and is virtually impossible for the reconstruction of ToLs. However, it would be interesting to study the gain and loss dynamics at different levels of the SCOP hierarchy such as the FSF and F levels of structural abstraction. We expect that patterns reported in this study will remain robust regardless of the SCOP conservation level and will extend the analysis to FSF in a separate publication.

We used maximum parsimony to search for the best possible tree and described the evolution of 420 free-living proteomes using the entire repertoire of 2,397 FFs as phylogenetic characters. We note that parsimony is most appropriate (and gives superior performance) for this kind of analysis as it performs better when the characters are evolving under different evolutionary rates [100]. Moreover, rescaling of raw abundance values into 24 possible character states considerably reduces the likelihood of convergent evolution. Reconstructing evolutionary history of species and studying domain emergence and loss patterns is a difficult problem complicated by a number of considerations (e.g. taxa and character sampling, biases introduced by organism lifestyles, ecological niches of organisms, and non-vertical evolutionary processes). We attempted to eliminate these problems by reconstructing whole-genome phylogenies, sampling conserved FF domains as characters, excluding parasitic and facultative parasitic organisms from study, and by using multistate phylogenetic characters. However, we realize that no method is free from technical and logical artifacts. Our analysis largely depends upon the accuracy of phylogenetic reconstruction methods, current SCOP domain definitions, reliability of function annotation schemes, and literature for organism lifestyle. However, we expect that recovered results will remain robust both with data growth and improvement in available methods and that drastic revisions to existing databases would be unlikely. For that reason we caution the reader to focus on the general trends and main conclusions of the paper (i.e. overwhelming gains and its consequences) rather than the actual numbers and discrepancies between the phylogenomic methods. Quantifying gain and loss events on a global scale is a difficult problem and our work lays foundations for more and improved studies in the future.

### Conclusions

We propose that grouping of protein domains into FFs provides a reliable character for a global evolutionary analysis that involves large number of proteomes. FF domains are both sufficiently conserved and informative to explore the many branches on the ToLs. The age and distribution of FFs in organismal groups is biased and carries the power to unfold superkingdom history and explain important structural and functional differences among superkingdoms. Based on our data, we propose the primacy of domain gains over losses over the entire evolutionary period, ongoing evolutionary adaptations in akaryotic microbes, evolution of emerging eukaryotic species by domain loss, an early origin for Archaea, and endosymbiosis leading to mitochondria as a crucial event in eukaryote diversification. Each of these conclusions is important for reconstructing the evolutionary past and predicting evolutionary events in the future.

## Supporting Information

Dataset S1 Names and classification of organisms used in phylogenetic analyses.
(XLS)

Dataset S2 Names, SCOP *css*, and the number of times each of the 2,262 parsimony informative FFs was gained/lost in *abundance* reconstruction. Eight FFs from the 420-proteome dataset were given new ids in SCOP 1.75 and were therefore renamed.
(XLS)

Dataset S3 Names, SCOP *css*, and the number of times each of the 2,249 parsimony informative FFs was gained/lost in *occurrence* reconstruction. Eight FFs from the 420-proteome dataset were given new ids in SCOP 1.75 and were therefore renamed.
(XLS)

Figure S1 Histograms displaying FF gain and loss dynamics for the phylogenetic trees rooted by the outgroup method. *Thermus thermophilus* (Deinococcus-Thermus) was used to root the archaeal tree while *Methanocaldococcus jannaschii* (Euryarchaeota) was used as outgroup for both Bacteria and Eukarya. The *x*-axes indicate evolutionary time (*nd*). Numbers in parenthesis represent the total number of taxa (proteomes) in each reconstruction, while *n* is the number of parsimony informative characters. Outgroup taxa were excluded from the calculations of gains and losses to eliminate any biases resulting from the artificial introductions of taxa into the dataset. Bars in red and blue indicate gains and losses respectively.
(TIF)
