# Supplementary figures and images for: Global Patterns of Protein Domain Gain and Loss in Superkingdoms
Source: PLoS Comput Biol. 2014 Jan 30;10(1):e1003452. doi: 10.1371/journal.pcbi.1003452 (PMC3907288; doi:10.1371/journal.pcbi.1003452)

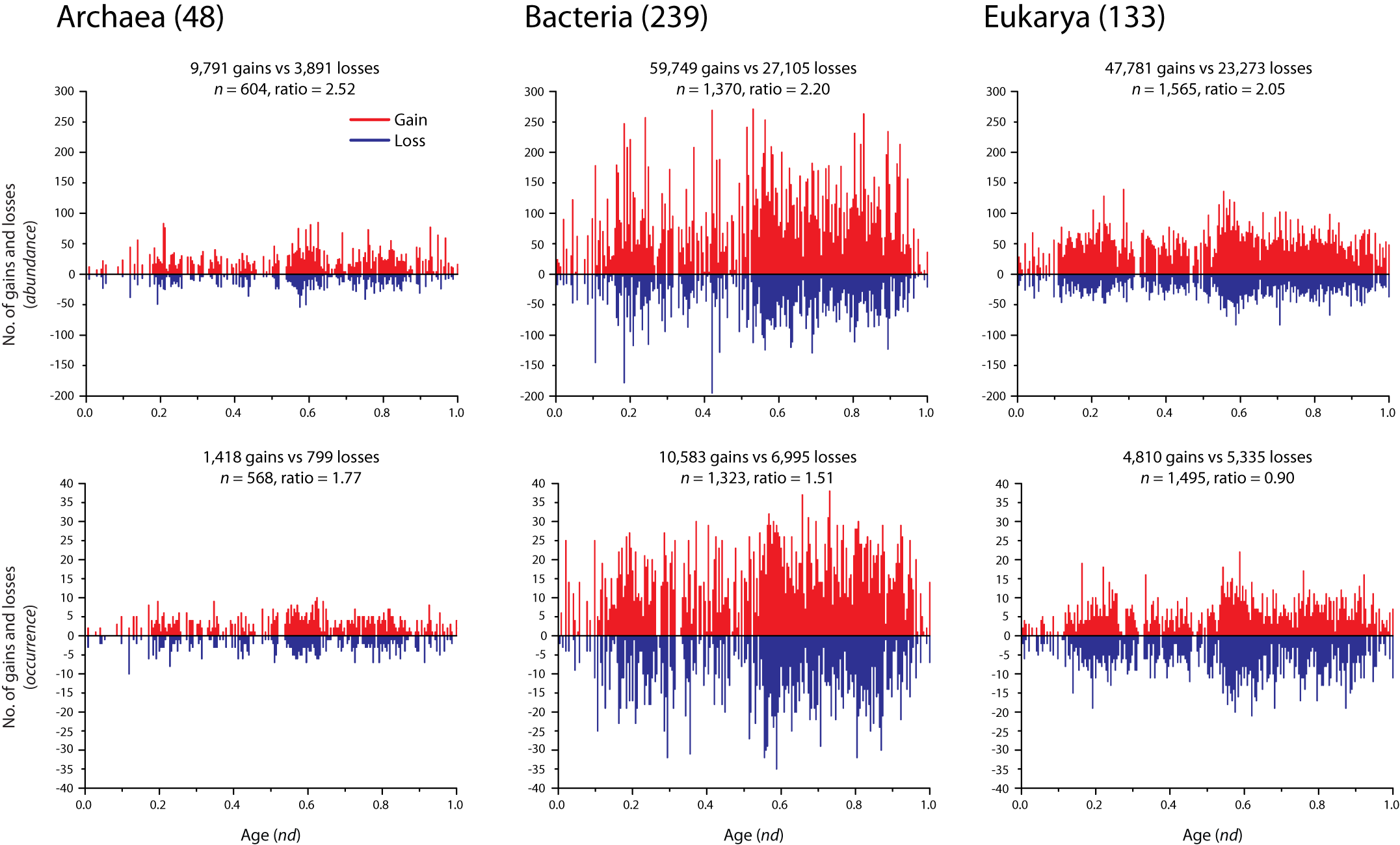

Supplement: Figure S1 — Histograms displaying FF gain and loss dynamics for the phylogenetic trees rooted by the outgroup method. Thermus thermophilus (Deinococcus-Thermus) was used to root the archaeal tree while Methanocaldococcus jannaschii (Euryarchaeota) was used as outgroup for both Bacteria and Eukarya. The x-axes indicate evolutionary time (nd). Numbers in parenthesis represent the total number of taxa (proteomes) in each reconstruction, while n is the number of parsimony informative characters. Outgroup taxa were excluded from the calculations of gains and losses to eliminate any biases resulting from the artificial introductions of taxa into the dataset. Bars in red and blue indicate gains and losses respectively. (TIF) [file pcbi.1003452.s004.tif]
